# Entrepreneurs’ Opportunities: From Exploratory learning to Entrepreneurial Improvisation

**DOI:** 10.1371/journal.pone.0342551

**Published:** 2026-02-17

**Authors:** Yi Hong, Ting Nie

**Affiliations:** School of Business, Macau University of Science and Technology, Macau, China; Covenant University, NIGERIA

## Abstract

**Purpose:**

In today’s highly dynamic environment, the ability of entrepreneurs to accurately recognize and quickly respond to opportunities plays an important role in their entrepreneurial performance. Entrepreneurial improvisation has gained wide attention. Based on the Stimulus-Organism-Response Theory, this study aims to examine the influence mechanism and boundary conditions of exploratory learning on entrepreneurial improvisation.

**Design/methodology/approach:**

Based on a two-wave survey of 303 members from five entrepreneurial platforms in mainland China, the dual moderating effects of environmental dynamism and risk-taking propensity on the relationship between exploratory learning and entrepreneurial improvisation were validated. Data analysis was conducted using SPSS 26.0, Amos 26.0, and Process 4.0 software, covering mediation effects, moderation effects, and moderated mediation effects.

**Findings:**

Opportunity identification mediated the relationship between exploratory learning and entrepreneurial improvisation. Environmental dynamism positively moderated the relationship between exploratory learning and entrepreneurial improvisation. Risk-taking propensity negatively moderated the relationship between exploratory learning and entrepreneurial improvisation.

**Research implications:**

Exploratory learning can facilitate entrepreneurial improvisation by enhancing opportunity identification. In a high dynamic environment, individuals with low risk-taking propensity are more likely to show higher entrepreneurial improvisation due to exploratory learning and opportunity identification.

**Originality:**

The study can contribute to a more comprehensive understanding of the formation and boundary conditions of entrepreneurial improvisational behaviors. It can also provide further empirical support for research on improvisation in entrepreneurship.

## 1. Introduction

As the uncertainty of the business environment strengthens, the traditional management philosophy based on planning and control has been seriously questioned. It has become a goal for organizations to pursue how to respond quickly to changes and seize fleeting opportunities in dynamic and complex environments [1,2]. Improvisation emphasizes the ability of individuals or organizations to respond quickly and effectively to unexpected situations, even without prior preparation or planning. It aims to utilize available resources at hand to seek solutions creatively [3]. Especially in the field of entrepreneurship, a complex environment influences entrepreneurs’ choices and decisions during the entrepreneurial process. Entrepreneurs are more likely to succeed in their entrepreneurial process if they can identify opportunities, implement improvisational behaviors, and cope with complex external environments. It will help to break through the existing barriers and continuously develop new products, technologies, or services to meet the expectations of the market, which will also be conducive to the improvement of entrepreneurial performance [4,5]. Entrepreneurial improvisation has thus gained widespread attention over the past decade [6,7].

The studies on entrepreneurial improvisation have developed rapidly in recent years, with scholars exploring its antecedents and consequences at multiple levels: individual, team, and organization respectively. Relational networks, human capital, environmental characteristics, cognitive inertia, and personal traits at the individual level significantly predict entrepreneurs’ improvisational behaviors [1,8–10]. Shared knowledge, resource piecing, and team communication at the team level [6,11–13] and organizational memory, information processing, internal resources, and organizational strategy at the organizational level have also confirmed the significant relationship with entrepreneurial improvisation [14–17]. Although there is some controversy about the consequences of improvisation, most studies have proven that entrepreneurial improvisation is often accompanied by novel outcomes that facilitate new knowledge and methods acquisition by organizations and individuals [18]. Moderate improvisation can increase organizational efficiency, flexibility, and entrepreneurial performance [4,5,19]. Current research on improvisation primarily emphasizes organizational and team levels. However, within the entrepreneurial context, the improvisational behavior of individual entrepreneurs plays a critical role. Improvisation involves creativity, and entrepreneurs need to create valuable, meaningful, and in-demand new products or services within a complex social system [7]. Creativity is an improvisational act of finding new solutions within a short period of time. Through improvisational behaviors, entrepreneurs can generate novel ideas, create new knowledge, and facilitate new ventures’ access to critical resources and investment opportunities [20,21]. Improvisation by entrepreneurs may also enhance new venture performance and contribute to entrepreneurial success [4,16]. The formation mechanisms and boundary conditions of individual entrepreneurial improvisation need to be further explored and empirically tested.

Stimulus-Organism-Response Theory indicates that external stimuli generate responses through an individual’s internal mechanisms that lead to specific behavioral outcomes [22]. Individuals’ traits and past experiences affect how they perceive, evaluate, and process stimuli, which in turn affects their behavioral responses. At the initial stage of entrepreneurship, learning is an effective way for entrepreneurs to absorb, accumulate and utilize knowledge [23]. The beginning of entrepreneurship is also the start of learning. In particular, exploratory learning, as a learning method based on self-directed exploration and discovery, emphasizes active participation and deep thinking on the part of the learner. Through exploratory learning, entrepreneurs can acquire new knowledge, understand new trends, and build new perspectives to identify existing or emerging business opportunities [24]. Entrepreneurs are generally highly sensitive to opportunities, and this external stimulus may trigger their strong entrepreneurial enthusiasm, prompting rapid strategic setting and resource allocation through improvisational behaviors [25]. In highly dynamic environments, entrepreneurs are more likely to take proactive actions as opportunities identified through exploratory learning may be fleeting. They will integrate existing cognition, emotions, and resources to respond spontaneously and immediately [1,10]. Entrepreneurship is a form of risk-taking behavior, where entrepreneurs need to invest time, resources, and money in exchange for a greater value goal. Entrepreneurs with a high propensity for risk are more willing to try new things and embrace new challenges [26,27]. They are more likely to be intrinsically motivated by the identified new opportunities, responding quickly and demonstrating high entrepreneurial improvisation. Both environmental dynamism and risk-taking propensity may be boundary conditions for the impact of exploratory learning on entrepreneurial improvisation through opportunity identification.

Based on Stimulus-Organism-Response Theory, this study aims to examine the formation mechanisms and boundary conditions of exploratory learning on individual entrepreneurial improvisation through a survey on members of five entrepreneurial associations in mainland China. The study contributes to the understanding of individual entrepreneurial improvisation by examining it from the perspective of learning stimuli. It empirically tests the dual moderating effects of environmental dynamism and risk-taking propensity using survey data from entrepreneurs. The findings not only offer practical insights for fostering improvisation in entrepreneurial contexts, but also enrich the empirical research on improvisational behaviors within the field of entrepreneurship.

## 2. Theory and hypotheses

### 2.1. Exploratory learning and entrepreneurial improvisation

Exploratory learning is a process where entrepreneurs actively reorganize and use their existing knowledge and skills to discover new approaches, develop original ideas, and create innovative solutions [28]. Entrepreneurs are constantly seeking new opportunities and developing new products, technologies and services to meet market needs through the linkage of resources and knowledge [29]. As a learning approach based on self-directed exploration and discovery, exploratory learning embodies a bottom-up learning process that emphasizes active participation and deep thinking [24]. It aims to develop new knowledge or build new capabilities. To enhance this learning process, entrepreneurs usually engage in practical experimentation: testing ideas in real-market conditions rather than relying solely on theoretical knowledge [26]. By using minimum viable products, entrepreneurs can release basic product versions to collect early user feedback. This approach validates their initial assumptions and enables them to pivot or refine their ideas to better meet market needs. Entrepreneurs need to abandon their practices and devote more to product innovation, thus laying the foundation for organizational value creation [30]. In highly competitive market environments with substantial uncertainty, it is increasingly difficult for entrepreneurial firms to sustain their competitive advantage. Innovation has become an important means of survival and growth for startups [31]. Exploratory learning is essential for organizations in dynamic environments to continuously innovate technology, and maintain sustainable growth [32]. Therefore, the integration of practical entrepreneurial insights into exploratory learning not only enriches the knowledge base of entrepreneurs but also equips them to innovate effectively and sustainably in an ever-evolving marketplace. This dual approach of experiential learning and strategic foresight lays a strong foundation for building successful, resilient businesses [33].

Improvisation is an individual’s spontaneous use of resources at hand with timely and creative intent in an unanticipated and turbulent environment [14]. It manifests the individual’s rapid response to unexpected events through quick adjustments in the way he or she works [3]. Entrepreneurs often encounter urgent issues with significant time constraints, limiting their ability to engage in extensive team discussions or detailed planning [34]. As a result, they frequently rely on improvisational behavior, which offers greater flexibility and adaptability in dynamic situations. It prompts entrepreneurs to go beyond their existing strategies and make quick judgments about new situations [25]. In practice, effective improvisation involves cultivating a mindset that embraces uncertainty [3]. Entrepreneurs can benefit from scenario planning: a technique used to visualize potential challenges and opportunities that may arise. By recognizing possible future scenarios, entrepreneurs can prepare themselves to act decisively and resourcefully when situations change unexpectedly [24]. Moreover, the dynamic nature of entrepreneurial environments often requires rapid shifts in resource allocation. This requires entrepreneurs to response to unexpected incidents or challenges [35]. Entrepreneurial improvisation implies the flexible use of existing resources to solve problems or capture new opportunities. It can play a crucial role in meeting unknown needs, driving organizational innovation, and accelerating the entrepreneurial process [4,5,20]. However, excessive improvisation can also lead to risks such as out-of-control, coordination difficulties, or over-reliance on intuition, which can result in financial losses [1,36]. While improvisation is an invaluable skill for entrepreneurs navigating turbulent environments, it must be complemented by strategic preparation, resource agility, and established protocols [31]. By applying these insights, entrepreneurs can leverage improvisation to navigate challenges more effectively.

Learning is a key element of entrepreneurship enhancement and an important driving force for entrepreneurs to achieve success [37]. Stimulus-Organism-Response Theory states that stimuli during the learning process have an impact on an individual’s internal mechanisms and behavioral responses [22]. Exploratory learning emphasizes active participation in self-directed exploration [38]. These external stimuli can promote the entrepreneur’s inquisitive spirit, improve problem-solving skills, and develop critical thinking. Entrepreneurs will then consider developing new products, adopting new technologies, or seeking new market opportunities, and take further actions in response to external stimuli [24]. The entrepreneur’s ability to gather resources, integrate knowledge, and utilize technology are all beneficial supports for their entrepreneurial improvisation [10,18]. Consequently, exploratory learning can help entrepreneurs continue to learn and grow, and develop innovative thinking and entrepreneurial skills so that they can be more flexible and confident in making decisions and taking actions during the entrepreneurial process. Research hypothesis 1 is proposed:

*Hypothesis 1: Exploratory learning positively influences entrepreneurial improvisation.*

### 2.2. The mediating role of opportunity identification

Opportunities are possible unknown market needs, underutilized resources or capabilities. Opportunity identification describes the process through which individuals identify novel, desirable, and feasible business ideas. It embodies an individual’s efforts to search and find business opportunities and is an essential component of any entrepreneurial process [25,39]. Only by identifying potential and valuable opportunities, entrepreneurs can have more possibilities of success [40]. Conversely, launching a business without a clear strategy not only fails to achieve the entrepreneurial goals, but also has the potential to result in serious financial losses. It is one of the most important competencies for a successful entrepreneur to identify and select appropriate entrepreneurial opportunities [41]. Entrepreneurs should be individuals who can recognize, assess, and capitalize on opportunities. The identification of opportunities serves as the fundamental basis of entrepreneurship [42].

As Stimulus-Organism-Response Theory indicates: Human behavior is a response to external stimuli acting on an individual’s internal mechanisms [22]. Exploratory learning is risky for innovative companies, but it is a powerful external stimulus for entrepreneurs. It helps entrepreneurs in market exploration and opportunity identification, which in turn triggers their subsequent behavioral exploration. Especially when entrepreneurial firms have bottlenecks in their development and are no longer able to gain a competitive advantage solely from the products they already have, entrepreneurs need to introduce new technologies or learn new knowledge to achieve their goals [25]. Exploratory learning plays a vital role in helping startups investigate market conditions, identify target customer segments, and develop new technologies. Without accurately identifying and acting on business opportunities, organizations cannot achieve sustainable growth. Thus, the process of opportunity recognition is essential to ensuring long-term corporate sustainability [42]. Improvisational behavior usually takes place in unexpected situations, environmental changes, or resource constraints [14]. It requires quick responses, creative thinking and flexibility. Improvisational behavior is an extension of opportunity identification. Research by Baker [43] has indicated that innovative decisions and operations (e.g., improvisational behavior) often rely on the precise identification and capture of complex information, which also involves the process of opportunity recognition [25]. When entrepreneurs become aware of a new opportunity, they tend to quickly adapt their current strategy or approach, and improvisational behavior becomes a means of realizing that opportunity. During this process, entrepreneurs not only need to make decisions at a very fast pace, but also use their creative thinking and problem solving skills to overcome any obstacles they may encounter [7,44]. Exploratory learning can trigger more frequent entrepreneurial improvisation through their opportunity identification. Research hypothesis 2 is proposed:

*Hypothesis 2: Opportunity identification mediates the relationship between exploratory learning and entrepreneurial improvisation*

### 2.3. Moderating role of environmental dynamism

Dynamic changes in the environment are manifested in increasing business risks. It is an objective situation faced by today’s organizations and poses a great challenge to their survival and development [45]. Uncertainty and volatility in the environment can lead to instability and changes in market demand. Enterprises need to constantly pay attention to changes and adjust their products or services in a timely manner to meet consumers’ needs [1,10]. New competitors may emerge quickly and existing competitors may increase their competition. To survive and develop in the fierce competition, enterprises must enhance their competitiveness and continuously commit to innovations and improvements [46]. For entrepreneurs, the environmental dynamism is more likely to bring them higher psychological pressure, and they need to assume greater responsibility and stress in the face of the uncertain future and risks [47]. Environmental dynamism increases the difficulty of recognizing opportunities, while resource-constrained startups often struggle to consistently identify and leverage these opportunities to achieve sustained performance [44,48]. By responding flexibly and immediately to changing circumstances, entrepreneurs can reduce risks, seize opportunities and ensure the survival of their start-ups [16]. In line with Stimulus-Organism-Response theory, highly dynamic environments intensify external stimuli, leading individuals to adapt their cognitive processes and respond more rapidly [22]. Exploratory learning can stimulate entrepreneurs’ innovative thinking and creativity. By learning new knowledge and skills, entrepreneurs can broaden the boundaries of their mindset and develop new ideas [24]. They may draw inspiration from different fields and discover new opportunities.

Entrepreneurship requires full consideration of the impact of changes in the external environment on the firm’s resources and capabilities [49]. The high dynamic environment is characterized by increased uncertainty. Market conditions, the political environment, and the economic situation become unstable [50]. The risks and challenges increase significantly due to the difficulty in predicting and controlling them, while the potential opportunities in the market tend to be less visible and harder to recognize [51]. For entrepreneurs, it is important to not only have keen market insights, but also to learn and observe changes in the market to better understand future trends and identify demand gaps [52]. In highly dynamic environments, opportunities are more likely to be recognized from exploratory learning. Entrepreneurs can be flexible and responsive to changes in circumstances and resources, adapting to market demands and increased competition [25]. Dynamic environments present risks and challenges, but also opportunities for innovation and change. At this time, entrepreneurs will also value the newly identified market opportunities more, flexibly adjust their entrepreneurial strategies and actions to win entrepreneurial success [20]. Therefore, research hypotheses 3 and 4 are proposed

*Hypothesis 3: Environmental dynamism positively moderates the relationship between exploratory learning and opportunity identification.*

*Hypothesis 4: Environmental dynamism positively moderates the indirect effect of exploratory learning on entrepreneurial improvisation through opportunity identification.*

### 2.4. Moderating effects of risk-taking propensity

Risk-taking propensity describes an individual’ s general inclination to engage in actions with uncertain outcomes, reflecting both their willingness to accept potential losses and their motivation to pursue opportunities that may offer significant rewards (Brockhaus, 1980). Risk propensity significantly influences how individuals behave under uncertainty. Those with a high willingness to take risks tend to focus on potential gains, whereas risk-averse individuals are more attentive to possible losses [53]. Entrepreneurs face a variety of risks during the entrepreneurial process. Not only do they need to face the various business risks involved in starting a business, but they may also have an impact on their personal and family finances as a result [54]. Compared to risk-averse leaders, entrepreneurs with a high risk propensity tend to focus on investments that are more risky and will be more engaged in corporate performance improvement and corporate ventures [55].

Entrepreneurs’ differences will also be reflected in their Stimulus-Organization-Response patterns in the entrepreneurial process. Risk-taking propensity, a common psychological tendency among entrepreneurs, often presents a stronger desire to invest when faced with high-risk projects with uncertain returns. They are exploratory and may be more willing to try new things and take on challenges [56]. Exploring new knowledge facilitates their identification of entrepreneurial opportunities. These individuals often satisfy their need for achievement through risky and innovative projects as a way of proving that they are more capable of managing and investing [26]. Meanwhile, high risk-averse individuals usually have high self-confidence and optimism. They believe in their own abilities and judgment with a positive attitude towards the future. This confidence and optimism makes them more courageous to try new things and face risks [27]. They are willing to take on challenges and high stress in anticipation of greater rewards and accomplishments [57]. For entrepreneurs with a high risk-taking propensity, they are more likely to acquire information and identify possible entrepreneurial opportunities through exploratory learning. They tend to make quick decisions to improve entrepreneurial performance. Therefore, research hypotheses 5 and 6 are proposed.

*Hypothesis 5: Risk-taking propensity positively moderates the relationship between exploratory learning and opportunity identification.*

*Hypothesis 6: Risk-taking propensity positively moderates the indirect effect of exploratory learning on entrepreneurial improvisation through opportunity identification.*

**Fig 1:**
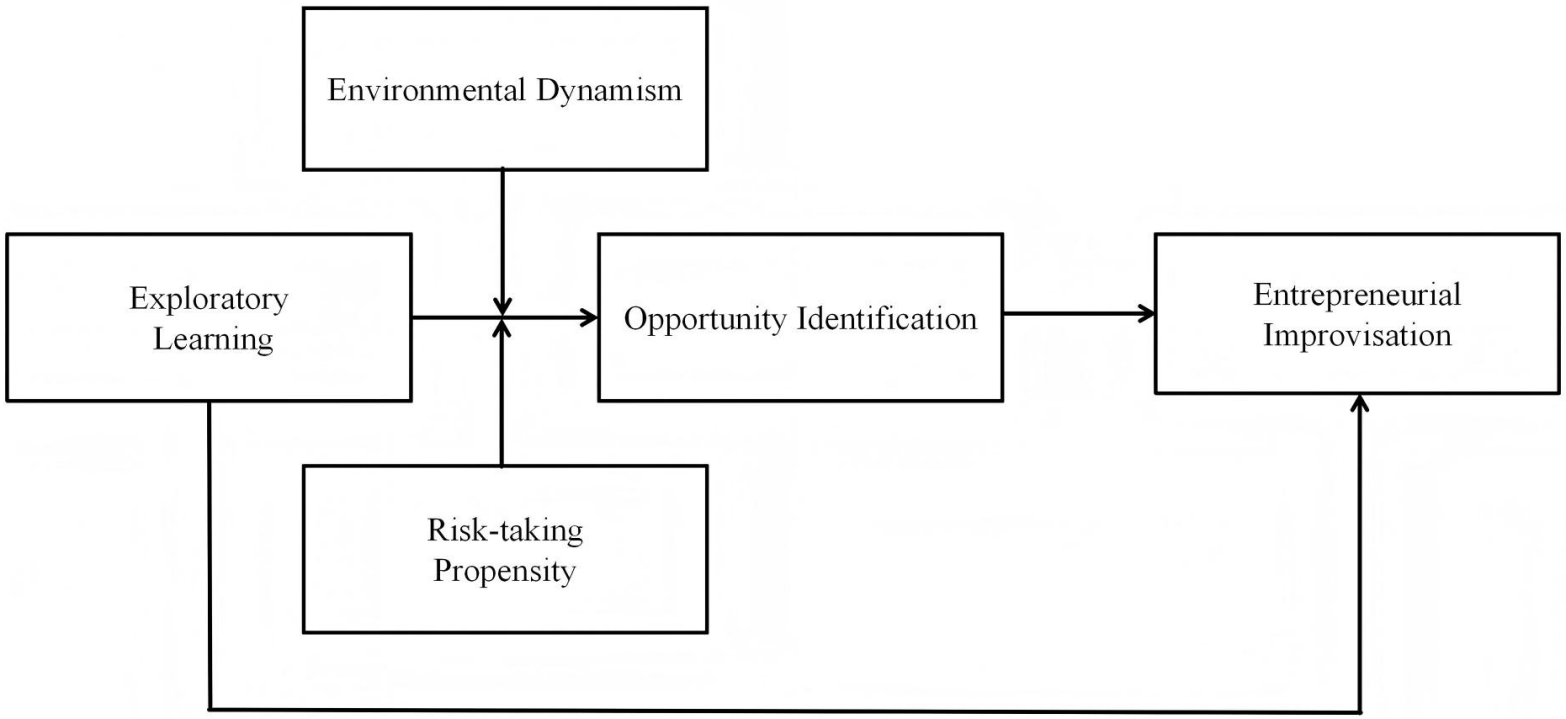
Theoretical framework.

The theoretical model is indicated Fig 1

## 3. Method

### 3.1. Procedure

Through questionnaire surveys, the study focused on exploring the influence mechanism and boundary conditions of exploratory learning on personal entrepreneurial improvisation. China’s current entrepreneurial environment is very active, with the number of entrepreneurs and entrepreneurial platforms steadily increasing. Through convenience sampling, this study selected five entrepreneurial platforms in China and collected information from all of their members. Entrepreneurs can receive assistance from entrepreneurial platforms during their ventures, including applying for and obtaining subsidies from local entrepreneurial policies, guidance on entrepreneurial skills and project analysis, provision of required entrepreneurial funding, as well as support for patent applications, project backing, and commercial services. Online questionnaires were distributed to entrepreneurship platform members through wjx.cn (one of the most influential online survey platforms in China). To reduce the impact of common method bias, data were collected in two phases (Oct. 2023-Jun. 2024). Each respondent participated two surveys with a month interval [58]. T1: Data on exploratory learning, risk taking propensity and environmental dynamism were collected based on entrepreneurs’ perceptions. T2: Data on opportunity identification and entrepreneurial improvisation were collected by matching phone number and gender. The study was reviewed by the Research Ethics Committee of the Business School of Macau University of Science and Technology. The methods in the study followed the Declaration of Helsinki. Written informed consent was obtained from all participants. All respondents were clearly aware of the purpose and process of the study with voluntary participation in the online survey.

### 3.2. Participants

The five entrepreneurial platforms were located in Shanghai, Guangzhou, Shenzhen, Beijing, and Foshan. These five cities have high population densities and large concentrations of entrepreneurs, which are the hubs for entrepreneurs in China’s information technology, manufacturing, finance, and service industries. We sent out survey invitations to all 865 members of the five entrepreneurial platforms. 545 members responded positively and participated in the survey. 303 valid questionnaires were ultimately collected. About 63% of the respondents were male and 37% female. Men were more likely to have a desire to start a business. Respondents are relatively young, with 77.9% of entrepreneurs under the age of thirty. The majority of respondents had received higher education, with 75.9% having a bachelor’s degree or above. The entrepreneurial field covered a wide range of industries, including manufacturing (30%), education and training (11.6%), the Internet (10.2%), cultural and entertainment (9%), Retail Industry (10%).Among these, the manufacturing, education and training, and internet sectors have the highest proportions.

### 3.3. Measurement

The survey was conducted on 5-point Likert scales, with 1 indicating complete disagreement and 5 indicating complete agreement.

**Exploratory Learning** was measured with a six-item scale adapted from Lubatkin (2006) [59]. The Cronbach’s alpha coefficient of this scale in the study is 0.843. The specific item is as follows: “I will look for creative ways to satisfy its customers’ needs.”

**Opportunity Identification** was measured with a six-item scale adapted from Ozgen Eren (2007) [60]. The Cronbach’s alpha coefficient of this scale in the study is 0.726. The specific item is as follows: “While going about day-to-day activities, I see potential new venture ideas all around me.”

**Entrepreneurial Improvisation** was measured with a six-item scale adapted from Magni (2009) [61]. The Cronbach’s alpha coefficient of this scale in the study is 0.921. The specific item is as follows: “When starting a business, I try new approaches to problems.”

**Environmental dynamism** was measured with a three-item scale adapted from Hoogh (2005) [62]. The Cronbach’s alpha coefficient of this scale in the study is 0.797. The specific item is as follows: “To what degree is your work environment dynamic.”

**Risk-taking propensity** was measured with a six-item scale adapted from Miller and Friesen(1982) [63]. The Cronbach’s alpha coefficient of this scale in the study is 0.743. The specific item is as follows: “I prefer to carefully analyze a situation before moving.”

### 3.4. Statistical analysis

SPSS 26, Amos26 and Process 4.0 were used to analyze the data, which mainly involved confirmatory factor analyses, correlation analyses, mediating effect test, moderating effects tests, and moderated mediation effects tests. Finally, a dual moderating effect was examined and discussed with the bootstrap test.

## 4. Results

### 4.1. Common method bias test

Confirmatory factor analysis (CFA) and Harman’s single-factor test were conducted to test the common method bias in the study as suggested by Podsakoff *et al.* (2003). Confirmatory factor analysis results indicated that the five-factor model (exploratory learning, opportunity identification, entrepreneurial improvisation, environmental dynamism and risk-taking propensity) had a better model fit than alternative models (χ^2^/*df* = 1.668, TLI = 0.949, CFI = 0.958, RMSEA = 0.047, RMR = 0.043). The model fit of the single-factor model was far from acceptable (χ^2^/*df* = 4.626, TLI = 0.722, CFI = 0.747, RMSEA = 0.110, RMR = 0.065). Harman’s single-factor test indicated that the variance explained by the first factor was 18.84%, which was much lower than the threshold level of 50% [58]. The findings suggested that the five-factor model had a good fit for the data, and no significant evidence of common method bias was found.

### 4.2. Descriptive statistical analysis

The correlation analysis results are shown in Table 1. When controlling for the impact of gender, age, education, and industry, exploratory learning was positively correlated with opportunity identification (r = 0.380, *p* < 0.01) and entrepreneurial improvisation (r = 0.614, *p* < 0.01); opportunity identification was positively correlated with entrepreneurial improvisation (r = 0.495, *p* < 0.01).

**Table 1:** Mean, Standard Deviation and Correlation Statistics (*n* = 303).

	Mean	SD	1	2	3	4	5	6	7	8
1. Gender	1.37	.484								
2. Age	2.91	.841	−0.101							
3. Edu	1.95	.691	0.045	−0.201^**^						
4. Industry	4.98	3.956	0.161^**^	−0.249^**^	0.140^*^					
5. EL	3.913	0.692	−0.093	0.011	0.145^*^	−0.039				
6. OI	3.515	0.534	−0.013	−0.051	0.195^**^	0.152^**^	0.380^**^			
7. EI	4.015	0.607	−0.099	−0.013	0.148^**^	−0.012	0.614^**^	0.495^**^		
8. RTP	3.424	0.616	−0.157^**^	−0.060	0.033	0.047	0.355^**^	0.281^**^	0.497^**^	
9. ED	4.085	0.694	−0.084	−0.084	0.115^*^	−0.054	0.522^**^	0.307^**^	0.556^**^	0.494^**^

** p < 0.01, * p < 0.05. EL: exploratory Learning; OI: opportunity identification; EI: entrepreneurial improvisation; ED: environmental dynamism; RTP: risk taking propensity.

### 4.3. Hypotheses testing

Hypothesis 1 presented that exploratory learning positively influences entrepreneurial improvisation. As shown in Table 2, the direct effect of exploratory learning on individual entrepreneurial improvisation was significant (b = 0.437, SE = 0.040, 95%CI [0.358, 0.517]). Hypothesis 1 is supported.

**Table 2:** Direct Effect and Indirect Effect (*n* = 303).

	Path	Effect	SE	LLCI	ULCI
Direct Effect	EL → EI	0.437	0.040	0.358	0.517
Indirect effect	EL → OI → EI	0.102	0.021	0.064	0.144

Note: EL: exploratory Learning; OI: opportunity identification; EI: entrepreneurial improvisation; LLCI: lower level of 95% confidence interval, ULCI: upper level of 95% confidence interval.

Hypothesis 2 presented that opportunity identification mediates the relationship between exploratory learning and entrepreneurial improvisation. As shown in Table 2, the indirect effect of exploratory learning on individual entrepreneurial improvisation through opportunity identification was significant (b = 0.102, SE = 0.021, 95%CI [0.064, 0.144]). Exploratory learning opportunities may promote individual entrepreneurial improvisation by increasing their opportunity identification. Hypothesis 2 is supported.

Hypothesis 3 and 4 presented that environmental dynamism and risk-taking propensity positively moderate the relationship between exploratory learning and opportunity identification. As shown in Table 3, the interaction term between exploratory learning and environmental dynamism was significant (b = 0.170, SE = 0.061, 95%CI [0.051, 0.289]). Environmental dynamism played a positive moderating role on the relationship between exploratory learning and opportunity identification. In a highly dynamic environment, the impact of exploratory learning on individual opportunity identification was stronger. Hypothesis 3 is supported. The interaction term between exploratory learning and risk-taking propensity was also significant (b = −0.175, SE = 0.074, 95%CI [−0.319, −0.030]). However, risk-taking played a negative moderating role on the relationship between exploratory learning and opportunity identification, which was different from the original hypothetical expectation. To individuals with low risk-taking propensity, the impact of exploratory learning on individual opportunity identification was stronger. Hypothesis 4 is not supported.

**Table 3:** Moderating Effect and Moderated Mediation Effect (*n* = 303).

	Moderating Effect						Mediated Moderating Effect			
Variable	Int_1	Effect	SE	*p*	LLCI	ULCI	Index	SE	LLCI	ULCI
	ED	0.170	0.061	0.005	0.051	0.289	0.059	0.034	0.001	0.130
	RTP	−0.175	0.074	0.018	−0.319	−0.030	−0.061	0.034	−0.132	−0.020

Note: ED: environmental dynamism; RT: risk-taking propensity; LLCI: lower level of 95% confidence interval, ULCI: upper level of 95% confidence interval.

Hypothesis 5 presented that environmental dynamism positively moderates the indirect effect of exploratory learning on entrepreneurial improvisation through opportunity identification, such that the relationship is stronger in a high dynamic environment (vs low). As shown in Table 3, the moderating effect of environmental dynamism on the indirect effect of exploratory learning on entrepreneurial improvisation through opportunity identification was significant (b = 0.059, SE = 0.034, 95%CI [0.001, 0.130]). The indirect influence of exploratory learning on entrepreneurial improvisation through opportunity identification was stronger in a high dynamic environment. Hypothesis 5 is supported.

Hypothesis 6 presented that risk-taking propensity positively moderates the indirect effect of exploratory learning on entrepreneurial improvisation through opportunity identification, such that the relationship is stronger for individuals with high risk-taking propensity (vs low). As shown in Table 3, the moderating effect of risk-taking propensity on the indirect effect of exploratory learning on entrepreneurial improvisation through opportunity identification was significant (b = −0.061, SE = 0.034, 95%CI [−0.132, −0.020]). The indirect influence of exploratory learning on entrepreneurial improvisation through opportunity identification was stronger for individuals with low risk-taking propensity, which was different from the original hypothetical expectation. Hypothesis 6 is not supported.

Considering the dual moderating effect of environmental dynamism and risk-taking propensity on the indirect effect of exploratory learning on entrepreneurial improvisation through opportunity identification, the conditional effects are shown in Table 4. When individuals were in a high dynamic environment (Mean+1SD) and with low risk-taking propensity (Mean-1SD), the indirect effect of exploratory learning on entrepreneurial improvisation through opportunity identification was stronger than the other three cases (b = 0.160, SE = 0.054, 95%CI [0.066, 0.274]). In a high dynamic environment, individuals with low risk-taking propensity were more likely to show entrepreneurial improvisation due to exploratory learning and opportunity identification.

**Table 4:** Conditional Effect (*n* = 303).

		ED - > OI					ED - > OI- > EI			
ED	RTP	Effect	SE	*p*	LLCI	ULCI	Effect	SE	LLCI	ULCI
L	L	0.223	0.051	0.000	0.122	0.324	0.078	0.022	0037	0.122
L	H	0.008	0.094	0.081	−0.177	0.192	0.003	0.040	−0.077	0.080
H	L	0.459	0.099	0.000	0.264	0.653	0.160	0.054	0.066	0.274
H	H	0.244	0.058	0.000	0.129	0.358	0.085	0.025	0.043	0.137

Note: EL: exploratory Learning; OI: opportunity identification; EI: entrepreneurial improvisation; ED: environmental dynamism; RT: risk-taking propensity; LLCI: lower level of 95% confidence interval, ULCI: upper level of 95% confidence interval.

## 5. Discussions

Through a survey on 303 members of China Entrepreneurship Platform, the study examined the influence mechanisms and boundary conditions of exploratory learning on entrepreneurial improvisation. The findings indicate that opportunity identification mediated the relationship between exploratory learning and entrepreneurial improvisation. Exploratory learning can facilitate personal entrepreneurial improvisation by enhancing opportunity identification. Environmental dynamism positively moderated the relationship between exploratory learning and entrepreneurial improvisation. Both direct and indirect effects of exploratory learning on entrepreneurial improvisation were stronger in highly dynamic environments. Risk-taking propensity negatively moderated the relationship between exploratory learning and entrepreneurial improvisation. The direct and indirect effects of exploratory learning on entrepreneurial improvisation were stronger for individuals with lower risk-taking propensity. In a high dynamic environment, individuals with low risk-taking propensity were more likely to show highest entrepreneurial improvisation due to exploratory learning and opportunity identification. The research hypotheses are partially validated.

### 5.1. Theoretical implications

In line with Stimulus-Organism-Response theory, this study verified the direct and indirect effects of exploratory learning on entrepreneurial improvisation, reaffirming that learning has an important value for entrepreneurs [22]. This is consistent with previous related studies [1,10,25,64]. Learning can not only help entrepreneurs to acquire a wide range of knowledge and skills, but also can provide an in-depth understanding of the market and industry they operate in. It will help entrepreneurs to better capitalize on business opportunities and develop strategies [4]. In particular, exploratory learning, as a learning style characterized by active exploration and self-directed learning, can enhance entrepreneurial competencies. Meanwhile, it serves as an external stimulus that inspires learners to actively discover knowledge and develop innovative thinking [24]. Entrepreneurs can identify and scrutinize new business opportunities and venture areas through self-directed exploration and practice. Along with increased flexibility and resilience, they are able to make quick decisions and actions in complex environments that provide a foundation for the growth of new ventures [10,64]. Exploratory learning facilitates entrepreneurial improvisational behavior through opportunity identification, which further validates Stimulus-Organism-Response Theory in the field of entrepreneurship. External stimuli generate responses through an individual’s internal mechanisms that lead to specific behavioral outcomes [22]. Meanwhile, this study also empirically complements existing entrepreneurial improvisation research by explaining the influence mechanisms of exploratory learning on entrepreneurial improvisation from the individual’s perspective.

In addition, this study verified the boundary effects of environmental dynamism and individual risk-taking propensity on the relationship between exploratory learning and entrepreneurial improvisation, which fully reflects Stimulus-Organism-Response Theory. Individuals’ traits and external environments affect how they perceive, evaluate, and process stimuli, which in turn affects their behavioral responses [22]. Through verification, The impact of exploratory learning on entrepreneurial improvisation through opportunity identification is stronger in high dynamic environments. This finding is consistent with most of the current research [3,7,10,12]. Improvisational behavior is highly situational and embodies a flexible way of responding based on situational changes [9]. Entrepreneurs perceive not only challenges but entrepreneurial possibilities in a dynamic environment. They are more sensitive to the uncertainty of the environment and are more likely to be motivated by the opportunities they discover through exploratory learning. They will thus display spontaneous and creative behaviors in response to emerging events [1]. Entrepreneurs can identify new business opportunities and venture capital fields through exploration and practice of environmental and behavioral experiences [4]. Exploratory learning is more likely to help entrepreneurs improve their cognitive abilities and identify entrepreneurial opportunities in a dynamic environment [65]. Existing research generally believes that entrepreneurs tend to be characterized by the propensity for risk-taking. They usually have a high tolerance for risk, which motivates them to keep trying to venture [26,27]. However, our study indicates that individual risk-taking propensity negatively moderated the relationship between exploratory learning and entrepreneurial improvisation. Individuals with a low risk-taking propensity are more likely to exhibit entrepreneurial improvisation due to exploratory learning and opportunity identification. Individuals’ attitudes toward risk are usually measured by their behavioral tendencies in dangerous situations. Personal attitudes toward risk depend on factors including prior knowledge and emotional responses to uncertainty [66]. Individuals with higher risk-taking tendencies pursue opportunities from internal drives. They are willing to take uncertainty and risk, dare to try new things and challenge traditional ideas, and will proactively seek out opportunities with high rewards [67]. Their entrepreneurial improvisation is essentially an intuitive response to their propensity for risk-taking, which does not necessarily require external stimuli. For entrepreneurs with a low risk-taking propensity, they are relatively cautious and are likely to respond only when they recognize the existence of opportunities. Their improvisational behavior is often a reflection of their response to external stimuli. Exploratory learning is more likely to act as a stimulus that motivates low risk-taking entrepreneurs to respond positively due to opportunity identification. In highly dynamic environments, entrepreneurs with low risk-taking propensity may have greater likelihood of engaging in improvisational behavior as a result of exploratory learning. The validation of the dual moderating effects of environmental dynamism and risk-taking propensity can further clarify the boundary conditions for the occurrence of improvisational behavior in the field of entrepreneurship.

### 5.2. Managerial implications

Entrepreneurs are keen thinkers and observers who are good at recognizing opportunities and seizing them. Meanwhile, they are also very cautious in facing risks. Especially when they have limited resources, they will control the risks to avoid bankruptcy. When the market is highly volatile, entrepreneurs tend to be conservative. In an uncertain market, blind investment is only a drain on resources. The study’s findings indicate that dynamic environments motivate low-risk entrepreneurs to adopt an exploratory learning approach to organize their thoughts and recognize opportunities. When the opportunity arises, they are prompted to achieve entrepreneurial success through improvisational behavior. After the epidemic, the negative side of the market became increasingly evident and a large number of entrepreneurs were caught in a difficult situation. Decision-making in a dynamic environment is characterized by speed and accuracy, and managing risk requires clear thinking and the ability to recognize precise opportunities. Entrepreneurs should take a more proactive approach to learning, especially in the midst of market ambiguity and constant change. They need to devote more time to acquiring and analyzing new trends in the market. Once a new investment opportunity is identified, immediate improvisation will help the entrepreneur to improve entrepreneurial performance and achieve commercial success. In the field of entrepreneurship, exploratory learning is a very effective way to get the latest information and opportunities, as well as an effective way for entrepreneurs to start their business attempts. Especially in a high dynamic environment, individuals with low risk-taking propensity are more likely to benefit from exploratory learning and opportunity identification. By actively promoting opportunities for continuous learning, adopting more agile practices, implementing structured risk assessment frameworks, and fostering diverse teams, entrepreneurs can maximize their potential for sustained improvisation and innovation in dynamic environments. This strategic approach not only enhances decision-making but also ensures that entrepreneurs are better equipped to capitalize on emerging opportunities.

## 6. Limitations and future studies

This study has the following limitations: the self-rating scales used in this study may lead to an overstatement of the impact of exploratory learning on individual entrepreneurial improvisation. Although the common method bias was proved to be not serious in this study through the two-stage survey and statistical tests, the risk of common method bias can be further controlled in future studies by considering paired surveys or adopting diverse research methods such as in-depth interviews and experimental studies [58]. All data in the study were obtained from members of entrepreneurial platforms in China. Chinese culture values stability and risk avoidance, and attitudes toward risk-taking are significantly different from those of many countries [68]. Therefore, the findings of this study may have some external validity issues, and future studies can explore the influence mechanisms and boundary conditions of exploratory learning on individual improvisational behaviors in different cultural contexts. Cross-cultural comparative studies will contribute to a more comprehensive explanation of entrepreneurial improvisation. In addition, this research explains the formation of individual entrepreneurial improvisation base on Stimulus-Organism-Response Theory. The theory emphasizes that external stimuli can trigger internal psychological changes in individuals and lead to behavioral responses. However, the improvisational behaviors of entrepreneurs are complex and dynamic. Key mechanisms at the team or organization level also need attention. The discussion from multiple perspectives such as macro-environment, organizational policies, leadership styles, and personal traits can help to gain a deeper understanding of entrepreneurial improvisation. And finally, the boundary conditions that transform entrepreneurial improvisation into entrepreneurial behaviors, as well as the entrepreneurial outcomes require further validation in the future.

## Supporting information

S1 File Dataset used in analysis. (XLSX)
